# Exploring the anti-inflammatory activities, mechanism of action and prospective drug delivery systems of tocotrienol to target neurodegenerative diseases

**DOI:** 10.12688/f1000research.131863.1

**Published:** 2023-03-27

**Authors:** Angela Maria Mathew, Saatheeyavaane Bhuvanendran, Rajesh Sreedharan Nair, Ammu K Radhakrishnan

**Affiliations:** 1 Department of Life Sciences, CHRIST (Deemed to be University), Bangalore, Karnataka, 560029, India; 2 Jeffrey Cheah School of Medicine and Health Sciences, Monash University Malaysia, Bandar Sunway, Selangor, 47500, Malaysia; 3 School of Pharmacy, Monash University Malaysia, Bandar Sunway, Selangor, 47500, Malaysia

**Keywords:** Tocotrienols, anti-inflammatory, neurodegenerative diseases, drug delivery, nanotechnology, vitamin E

## Abstract

A major cause of death in the elderly worldwide is attributed to neurodegenerative diseases, such as AD (Alzheimer’s disease), PD (Parkinson’s disease), ALS (Amyotrophic lateral sclerosis), FRDA (Friedreich’s ataxia), VaD (Vascular dementia) etc. These can be caused due to multiple factors such as genetic, physiological problems like stroke or tumor, or even external causes like viruses, toxins, or chemicals. T3s (tocotrienols) exhibit various bioactive properties where it acts as an antioxidant, anti-inflammatory, anti-tumorigenic, and cholesterol lowering agent. Since T3 interferes with and influences several anti-inflammatory mechanisms, it aids in combating inflammatory responses that lead to disease progression. T3s are found to have a profound neuroprotective ability, however, due to their poor oral bioavailability, their full potential could not be exploited. Hence there is a need to explore other drug delivery techniques, especially focusing on aspects of nanotechnology. In this review paper we explore the anti-inflammatory mechanisms of T3 to apply it in the treatment of neurodegenerative diseases and also discusses the possibilities of nano methods of administering tocotrienols to target neurodegenerative diseases.

## Introduction

Initially known as “an anti-sterility factor, X,” vitamin E was first discovered in 1922 by Evans and Bishop, as it positively aided reproduction. ^1^ Vitamin E consists of eight isoforms, namely, α, β, γ, δ - tocopherols and α, β, γ, δ - tocotrienols. The Tphs (tocopherols) and T3s (tocotrienols) contain a chromanol ring (bicyclic phenols) and hydrocarbon side chain, wherein Tphs have an aliphatic saturated phytyl tail and T3s have an unsaturated farnesyl tail. ^2^

Like other lipophilic vitamins, vitamin E absorption depends on a fat-rich diet, bile salts, and pancreatic enzymes. When supplemented orally, vitamin E homologs are absorbed *via* the mesenteric lymph nodes in the intestine as chylomicrons, a huge lipoprotein containing triglycerides composed of lipids from cholesterol and fatty acids, that either enter the tissues or the liver. The lipoprotein lipase (usually found in the adipose and muscle tissue) enzyme, bound to the endothelial cells of the capillaries, hydrolyses these vitamin E chylomicrons and aids in the transportation of vitamin E and other lipids to the tissues. Most of the vitamin E in the chylomicron remnants is assimilated into VLDL (very-low-density lipoprotein) in the liver, and the remaining is eliminated in the bile secretions. Cytochrome P450 metabolizes vitamin E through hydroxylation and oxidation of its side chains. The end product metabolites are eliminated in feces and urine, namely, CMBHC (carboxymethylbutyl hydroxychromans) and CEHC (carboxyethyl hydroxychromans). ^3^

While drawing a comparison between the Tphs and T3s, it can be found that the former has a better biopotency than the latter, with respect to the biological half-life (t _1/2_), which is in turn linked to its side chain tail length. Due to its unsaturated carbon tail, T3 is metabolized, degraded, and excreted in the urine quicker than Tph. Human cytochrome P450 (called CYP4F2) catalyzes ω-hydroxylation, and Tph and T3 are converted to carboxychromanols. Numerous factors contribute to the degradation of vitamin E isoforms, including their respective molecular structures, the number of methyl groups attached to the chromanol ring, and the stereochemistry of the carbon tail. The intracellular metabolic breakdown of vitamin E isomers was found to be catalyzed by only the CYP4F2 enzyme (t _1/2_). ^4^ Due to its unsaturated isoprenoid carbon tail, the T3 tail is shorter than Tph. This property of T3 is also found to have many superior bioactivities than Tph, which will be discussed in the following sections.

The functional aspect of T3 can provide neuroprotection against oxidative stress induced by free radicals, resulting in defective synapses and neuronal damage. The structure and functionality of the neurons were sustained upon T3 administration, which was evaluated based on their neuroprotective properties against neurotoxic metabolites involved in diminishing cognitive functions, like NO (nitric oxide) and glutamate-induced ROS (reactive oxygen species). It is found that orally supplemented T3 can enter systemic circulation and is delivered to the brain and cerebrospinal fluid by crossing the BBB (blood–brain barrier). ^4^

DNA chip analysis found that T3 downregulated the gene expression of PKC (protein kinase C) in HUVEC (human umbilical vein endothelial cells), exhibiting an anti-telomerase activity and downregulating VEGF (vascular endothelial growth factor) receptor in endothelial cells, thus proving its ability to inhibit angiogenesis. ^5^ Although vitamin E isomers were found to have potential anticancer activities due to their low solubility (in aqueous media) and bioavailability when administered orally, *in vivo* studies in animals are often affected. Vitamin E formulations in solvents like DMSO (dimethyl sulfoxide), ethanol, and vegetable oil emulsions, also have a disadvantage in clinical trials owing to their hydrophobic nature. ^6^

In this review, we focus on exploring the anti-inflammatory property of T3 and its application to treat progressive neurodegenerative diseases, alongside discussing the various drug delivery techniques of T3 to ensure greater bioavailability.

### Tocotrienol

Vitamin E is a lipophilic vitamin, which has eight naturally occurring isoforms, namely, α, β, γ, δ - tocopherol and α, β, γ, δ - tocotrienol. The α-Tph has been studied the most because of its increased bioavailability from the diet, and also it is found in almost every cell in the body. Due to the presence of α-Tph, the dietary uptake of T3 homologs is usually reduced, leading to less bioavailability *via* oral administration. Majorly due to this reason, for many years, researchers have been focusing more on the properties of α-Tphs. Even the absorbed β, γ, δ-Tphs are eliminated through the bile secretions and finally removed *via* feces, whereas the α-Tph is excreted through the urine. ^7^ But in recent years, it has been found that T3 has more potent antioxidant and anti-inflammatory properties. All these will be discussed in the later sections of this review.

**Chemical aspect**

**Figure 1.**
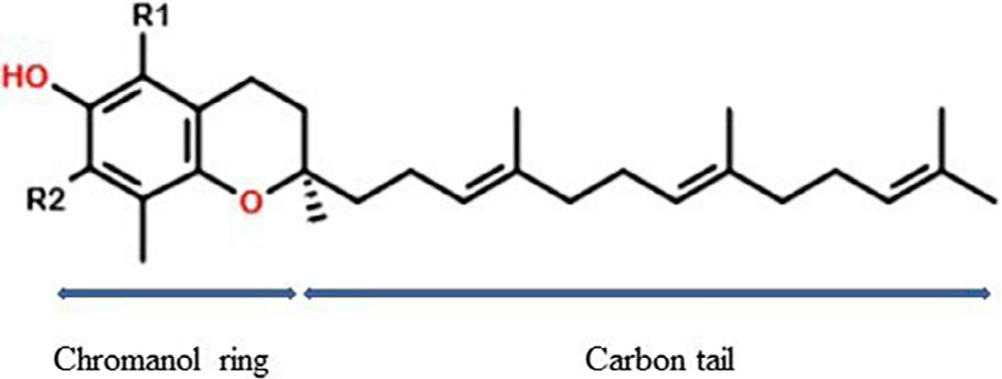
Tocotrienol chemical structure.

Vitamin E is a hydrophobic/lipophilic phenolic compound. Vitamin E isomers, namely Tph and T3, are known as tocochromanols. They have a chromanol ring and a phytyl tail ( Figure 1). T3 has a farnesyl tail with three double bonds at carbon 3, 7, and 11 and has four isoforms, α, β, γ, and δ-T3, based on the position of the methyl group on the chromanol ring. On the other hand, Tph has a saturated phytyl tail; this difference in the long carbon tail accounts for the varied biological activities of Tph and T3. The β-T3 and γ-T3 are structural isomers and contain the same number of the methyl group on the chromanol ring. In comparison to β-T3 and γ-T3, α-T3 has an extra methyl group and δ-T3 has one less methyl group ( Table 1). ^8^

**Table 1.** Tocotrienol subtypes.

Name	R1	R2
α-T3	**CH**_**3**_	**CH**_**3**_
β-T3	**CH**_**3**_	**H**
γ-T3	**H**	**CH**_**3**_
δ-T3	**H**	**H**

**Functional aspect**

Tocotrienol has numerous pharmaceutical applications such as antioxidant, ^9^ anti-inflammatory, ^10^ anti-tumorigenic, ^11^ hypolipidemic, ^12^ stimulates an immune response, ^13^ cardiovascular health, ^14^ bone health ^15^ and neuroprotection. ^16^ The properties of T3 that can be attributed to treating neurodegenerative diseases can be its antioxidant, cholesterol-lowering, and anti-inflammatory nature. ^17^

### Anti-inflammatory property

A major contributing factor towards the pathogenesis of many diseases such as atherosclerosis ^18^ and other cardiovascular diseases, ^19^ rheumatoid arthritis, ^20^ osteoporosis, ^21^ diabetes mellitus, ^22^ COPD (chronic obstructive pulmonary disease), ^23^ Alzheimer’s disease, ^24^ and cancer, ^25^ is inflammation. The inflammatory responses are mediated by leukotrienes and prostaglandins, whose precursor is arachidonic acid. ^26^^,^^27^ Cyclooxygenase (COX) 1 and 2 catalyze the oxidation of arachidonic acid to synthesize prostaglandin E2 (PGE2), ^27^ which is involved in the production of cytokines. ^28^ Leukotriene B4, another derivative of arachidonic acid catalyzed by the 5-lipoxygenase (5-LOX) enzyme, is a strong chemotactic agent. ^26^ Pro-inflammatory cytokines have been implicated in the pathogenesis of inflammation-related diseases. Central transcription factors, such as NFκB (nuclear factor κ-B) and JAK-STAT6/3 (Janus kinase/signal transducers and activators of transcription), facilitate the gene expression of numerous pro-inflammatory cytokines. ^29^

TRF (tocotrienol rich fraction) from palm oil has prominent anti-inflammatory activity by blocking NFκB pathway activation and can selectively inhibit COX-2 gene expression. The COX-2 downregulation also resulted in the consequent suppression of PGE2 production. This work also describes that TRF can effectively inhibit LPS (lipopolysaccharides)-induced NO production, and secretion of inflammatory cytokines like IL-4, IL-8, TNF-α and iNOS (inducible nitric oxide synthase) in a dose-dependent manner in LPS-stimulated human monocytic THP1 cells. ^30^ T3, inhibiting NFκB and mTOR (mammalian target of rapamycin) suppresses the SASP (senescence-associated secretory phenotype) produced by senescent cells or even selectively removes these aged cells through senolysis (selective lysis of senescent death). ^10^

δ-T3 has a rare dual biological property, which was the first to be discovered in any naturally-occurring compound to possess this characteristic, where it has both anti-inflammatory (inhibition) and pro-inflammatory (activation) properties based on its concentration. ^31^

For the first time, it was reported that γ-T3 could lower the inflammation and oxidative stress induced by cigarette smoking via enhancing Nrf2 (nuclear factor erythroid-2-related factor 2) activation and inhibiting the nuclear translocation of pro-inflammatory transcription factors like NFκB and STAT3. ^32^ γ-T3 upregulates A20 (inhibitor of NFκB) and thereby inhibits the TNF-α-induced activation of NFκB *via* modulating the sphingolipid pathways. ^33^

A study conducted in the FeNTA (ferric nitrilotriacetate) model reported that palm-oil-derived tocotrienol is more potent in protecting against bone resorption, eventually leading to bone loss and osteoporosis. It was found that the palm-oil tocotrienol mixture significantly lowered the levels of pro-inflammatory cytokines such as IL-1 and IL-6 caused by FeNTA toxicity. ^34^ It was also suggested that this property could be because of its antioxidant activity.

δ-T3 can decrease the production of pro-inflammatory cytokines like IL-6 and MCP-1 (monocyte chemoattractant protein) and consecutively increase the secretion of anti-inflammatory cytokines like IL-10, ultimately lowering inflammation and improving lipid metabolism. In addition, it also reduced liver triglycerides, macrophage infiltration, and adipocyte size, resulting in an overall better metabolically healthy profile. ^35^ *d*- *δ*-T3 can mediate the downregulation of PPAR-γ (peroxisome proliferator-activated receptors) leading to reduced levels of triglycerides within the cells, followed by decreased uptake of glucose and lower levels of proteins such as HMG CoA (hydroxyl-methyl-glutaryl co-enzyme A) reductase, p-Akt (PKB - Protein kinase B) and GLUT-4. ^36^ In LPS-stimulated macrophages, δ-T3 can prevent inflammation by inhibiting LPS-stimulated NO, suppressing NFκB and AP-1, and also inhibiting the phosphorylation of JNK (c-Jun N-terminal kinase) and ERK1/2 (extracellular regulated protein kinases), and proinflammatory cytokines (IL-1β, IL-6, IFN-γ, TNF-α). Altogether δ-T3 inhibits the MAPK (mitogen-activated protein kinase) and PPAR-activated signaling. ^37^

γ-T3 can delay the onset of diabetes mellitus by inhibiting NLRP3 (NOD-, LRR- and pyrin domain-containing protein 3) inflammasome ^38^ and has anti-adipogenic action via AMPK (5′ AMP-activated protein kinase) and autophagy activation. ^39^ γ-T3 downregulates the C/EBP-β and upregulates C/EBP-α, contributing to the inhibition of adipocyte differentiation at an early phase. ^39^ By suppressing NFκB activation, γ-T3 could attenuate the surged IL-6 and MCP-1 secretion caused by TNF-α induced inflammation. It regulates adiponectin secretion via PPAR-γ gene expression in adipocytes. ^40^ The suppression of NFκB signaling by T3 isomers leads to the halting of tissue inflammation and can be credited to its anti-inflammatory property.

**Figure 2.**
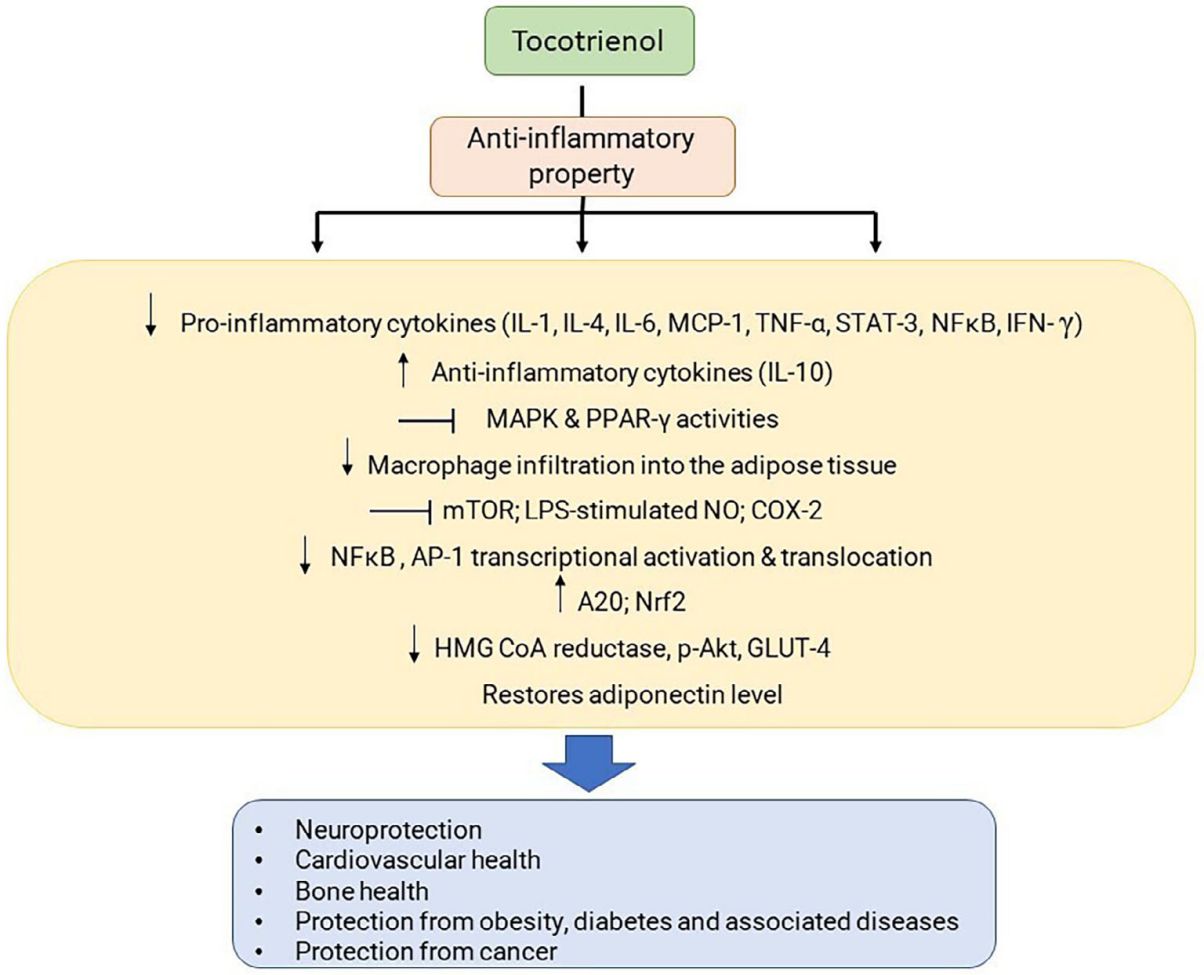
Anti-inflammatory activities of T3. Abbreviations: IL - Interleukin, MCP - Monocyte chemoattractant protein, TNF - Tumor necrosis factor, NFκB - Nuclear factor κ-B, IFN - Interferon, MAPK - Mitogen-activated protein kinase, PPAR - Peroxisome proliferator-activated receptors, mTOR - Mammalian target of rapamycin, LPS - Lipopolysaccharide, NO - Nitric oxide, COX - Cyclooxygenase, AP - Activator protein, Nrf2 - nuclear factor erythroid-2-related factor 2, HMG Co A - hydroxyl-methyl-glutaryl co-enzyme A.

**Figure 3.**
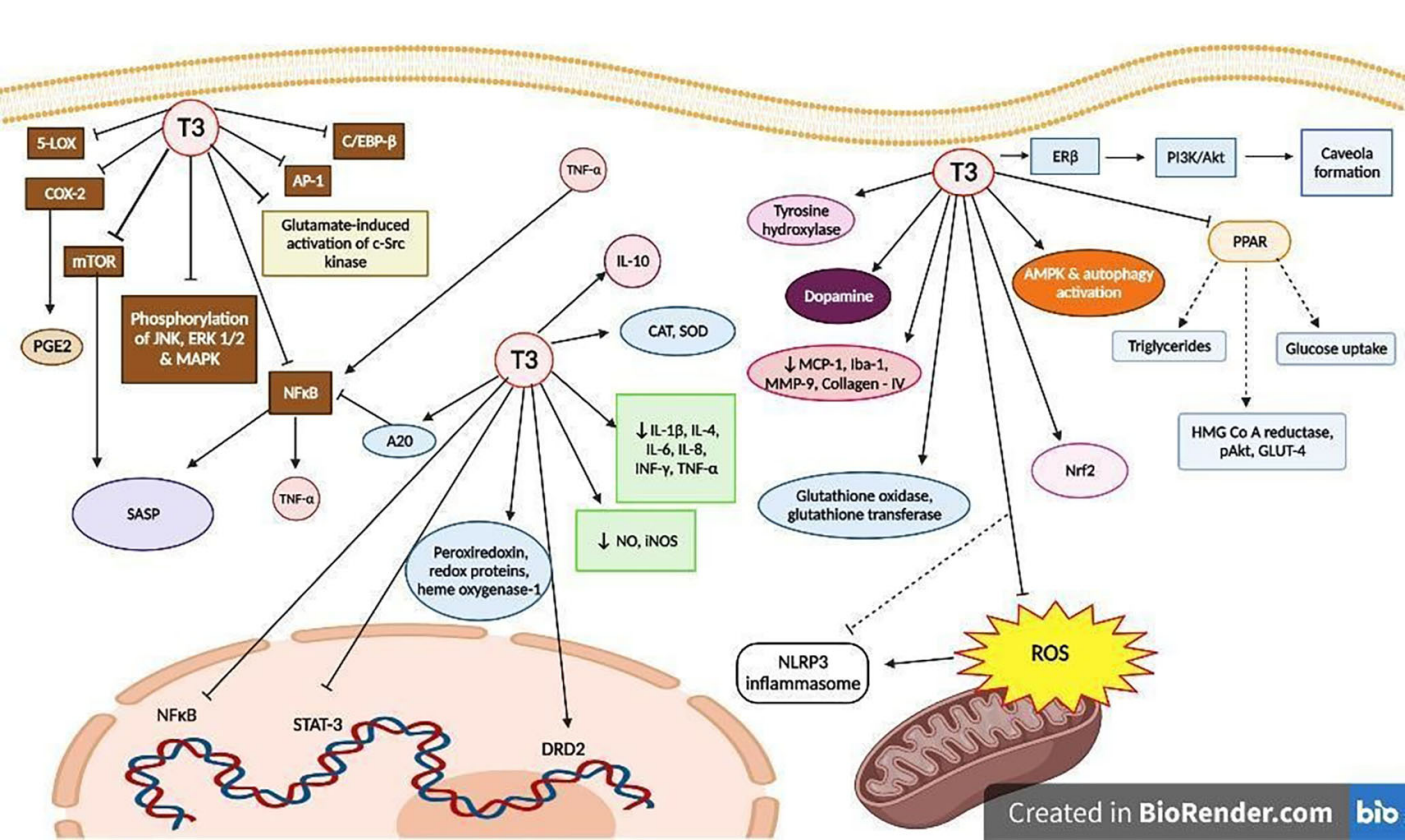
Anti-inflammatory molecular mechanisms involving T3. Abbreviations: LPS - Lipopolysaccharide, NO - Nitric oxide, TNF - Tumor necrosis factor, COX - Cyclooxygenase, LOX - Lipoxygenase, NFκB - Nuclear factor κ-B, iNOS - Inducible nitric oxide synthase, AP - Activator protein, PGE2 - Prostaglandin E2, IL - Interleukin, Nrf2 - nuclear factor erythroid-2-related factor 2, IFN - Interferon, SOD - Super oxide dismutase, CAT - Catalase, ROS - Reactive oxygen species, HMG Co A - hydroxyl-methyl-glutaryl co-enzyme A, SASP - senescence-associated secretory phenotype, JAK/STAT - Janus kinase/signal transducers and activators of transcription, MCP - Monocyte chemoattractant protein, NLRP3 - NOD-, LRR- and pyrin domain-containing protein 3, AMPK - 5' AMP-activated protein kinase, PPAR - Peroxisome proliferator-activated receptors, JNK - c-Jun N-terminal kinase, ERK - Extracellular regulated protein kinases, MAPK - Mitogen-activated protein kinase, mTOR - Mammalian target of rapamycin, c-Src – cellular Src, MMP - Matrix metalloproteinase, Iba - Ionized calcium-binding adapter molecule.

Another important aspect that needs to be addressed is “whether the anti-inflammatory and anti-oxidant properties go hand-in-hand?”. The antioxidant and anti-inflammatory ability of T3 are intrinsically linked through numerous mechanisms since inflammatory response accompanies oxidative stress. ^41^ Chronic tissue inflammation can be accounted for by the oxidative stress produced within cells due to cellular damage caused by ROS. Both antioxidant and anti-inflammatory signaling pathways complement each other since antioxidants alleviate oxidative damage by scavenging free radicals. A bioactive compound with an antioxidant effect will undoubtedly be an anti-inflammatory. Vitamin E homologs are popular dietary supplements, especially due to their antioxidant activities, wherein they prevent inflammatory responses in the cellular microenvironment caused by endogenous and exogenous oxidative agents. T3, a non-enzymatic antioxidant compound, can influence the enzymatic counterparts, including enzymes like SOD (super oxide dismutase), glutathione oxidase, CAT (catalase), peroxiredoxin and redox proteins, heme oxygenase-1, and glutathione transferase. ^4^ In vitamin E isoforms, the antioxidant property is based on the number of hydroxyl groups, and the T3 has a superior antioxidant property than Tph, which is in the order α > β > γ > δ, and can be credited to their greater distribution in the plasma membrane bilayer and interaction with lipid peroxyl radicals. ^2^ Figure 2 and Figure 3 demonstrate the anti-inflammatory properties of T3.

### Neurodegenerative diseases and tocotrienol

T3 has superior biological properties than Tph, with prominent antioxidant and anti-inflammatory effects. T3 can hinder the mevalonate pathway suppressing cholesterol biosynthesis. ^42^ Hepatic HMG CoA reductase inhibition can be attributed to an unsaturated hydrocarbon side chain. ^43^ T3 downregulates the HMG Co-A reductase due to this hypolipidemic and anti-inflammatory ability implicated in the medical condition of AD. ^44^

The tissue-specific macrophages called the microglia are involved in the neurogenesis and homeostasis in the CNS, which react to various stimuli *via* the secretion of inflammatory cytokines. These inflammatory cytokines can be considered a warning sign for identifying the occurrence of neuro-diseases like AD, PD, ALS, stroke, multiple sclerosis, *etc.* δ-T3 derived from palm oil attenuates NO production, IL-1β expression, and 5-LOX mRNA expression in BV2 microglia. It also inhibited the expression of PGE2 and selective inhibition of COX-2 and not COX-1. ^45^ The neuroprotective effects of various T3s are listed out in Table 2.

**Table 2.** T3 treatment used for various neurodegenerative diseases.

References	Disease condition	Chosen subjects	Dosage	Treatment period/mode	Results	
^50^	**AD**	SH-SY5Y neuroblastoma cells & N2a (neuro 2a) cells	α-Tph & α-T3 (10 μM each)	24 h	α-Tph	↓ ROS; ↓ lipid peroxidation; ↓ cholesterol
					α-T3	↓ ROS; ↓ lipid peroxidation; ↓ cholesterol (pronounced effect); ↑ Aβ production; ↓ degradation of Aβ
^98^		Male B6C3-Tg mice (Double transgenic)	TRF, α-Tph (200 mg/kg each)	6 months (Oral)	↓ SOD; ↓ DNA damage; ↑ CAT	
^99^		Sprague-Dawley rats	T3 (100 mg/kg)	8 weeks (Oral)	↓ F2-IsoPs suggesting antioxidant property; ↑ pyramidal cell viability	
^48^		Male B6C3-Tg mice (Double transgenic)	TRF (200 mg/kg)	6 months (Oral)	↑ antioxidant activity; ↑ neuroprotection; ↑ stability of DNA; ↑ motor learning & spatial memory; ↓ ROS production; ↓ αβ induction; ↓ neuronal apoptosis; ↓ amyloid plaque aggregation	
^52^	**PD**	SH-SY5Y neuroblastoma cells	α, β, γ, δ-T3 1 μM	48 h	γ-T3, δ-T3 involved in activation of ERβ/PI3K/Akt signaling via caveola formation (this effect is not accounted for antioxidant property of T3)	
^53^		C57BL/6 mice MPTP-induced	δ-T3 100 μg/kg	Day 2-6 (Oral gavage)	δ-T3 inhibited loss of SN neurons; ↑ motor activity	
^16^		Male Sprague Dawley rats	α-T3, γ-T3 10 mg/kg	28 days (Oral)	↓ motor deficit; ↑ neural functions via ↓ inflammation, revert neuronal degradation; preserved SN & STR fiber	
^100^		SH-SY5Y neuroblastoma cells	TRF (α, γ, δ-T3) 0.1 μg/mL	24 h	↑ cell viability; ↑ dopamine; ↑ TH; ↑ endogenous antioxidant enzymes like SOD, CAT; scavenge ROS	
					α-T3	Prevent leakage of α-synuclein
					γ-T3	↓ apoptosis
					δ-T3	↑ DRD2 gene expression
^101^	**Ischemic stroke**	C57BL/6 male mice	α-T3 50 mg/kg	10 weeks (Oral)	Able to revert the loss of miR-29b against stroke; decreased lesion size	
^102^		Male ICR mice brain under tMCAO for 1 h	Tocovid (α-Tph + TRF) 200 mg/kg/day	30 days (Oral)	↑ Neurobehavior; ↓ infarct volume; ↓ post-stroke injury; ↓ TNF-α, MCP-1 and Iba-1 (inflammatory markers); ↓ MMP-9, collagen IV and IgG (neurovascular units)	
^58^	**FRDA**	Humans of age groups 18-45 years	EPI-743 (α-T3 quinone) 200 mg or 400 mg	24 months (12+12) (Oral – 3 times/day)	↑ neurological functioning	
^59^		Humans	Idebenone + T3 mixture 5 mg/kg/day	12 months	Oxidative stress and inflammation parameters showed normal values; ↑ CMR; ↓ myocardial damage; ↑ Nrf2	
^60^	**VaD**	Male Sprague Dawley rats	TRF 30, 60 & 120 mg/kg	21 days (Oral)	↑ PDGF-C; prevented memory loss; ↓ insulin resistance; ↓ neuronal damage; ↑ cerebrovascular functioning; ↑ cholinergic activity	

Abbreviations: ROS - Reactive oxygen species, Aβ - Amyloid β, SOD - Superoxide dismutase, CAT - Catalase, F2-IsoPs - F2-isoPs - F2-isoprostanes, SN - Substantia nigra, STR - Striatum, TH - Tyrosine hydroxylase, TNF - Tumor necrosis factor, MCP - Monocyte chemoattractant protein, MMP - Matrix metalloproteinase, Iba - Ionized calcium-binding adapter molecule, CMR - Cardiac magnetic resonance, PDGF-C - Platelet-derived growth factor-C.

**AD**

AD is a neurodegenerative illness that is manifested as a progressive dementia leading to cognitive and memory impairment and various other biological changes such as Aβ (amyloid β) plaque accumulation, neurofibrillary entanglement, synaptic loss, to name a few. High cholesterol levels are linked to the pathogenesis of AD. A surge in cholesterol levels and cholesterol biosynthesis will lead to proteolytic cleavage of APP (amyloid precursor protein) by β-secretase and γ-secretase to produce Aβ. ^46^

The anti-inflammatory activity of T3 can be attributed to its protection against AD. ^47^ TRF could successfully hinder the progression of AD by regulating multiple genes and signaling pathways involved in the pathogenesis. ^48^ Ibrahim *et al*. ^49^ found that when administered at higher concentrations, α-Tph was able to decrease the Aβ aggregation, whereas, α-T3 even at lower concentrations was able to both reduce the Aβ aggregation and disaggregate the already formed Aβ fibrils. γ-T3, on the other hand, exhibited the ability to reduce the Aβ oligomerization, apart from lowering the Aβ aggregation and disaggregation of Aβ fibrils. ^49^

Alongside the positive effects of T3, it is also found to have some negative impacts on AD. α-Tph and α-T3 tend to enhance the production of Aβ ( *via* β-secretase and γ-secretase production) and decrease the degradation of Aβ, thereby facilitating the accumulation of plaques. ^50^ When vitamin E alone had the potential to increase p-Akt, lower oxidative stress via decreasing ROS production that occurs as a result of insulin resistance, and reduce AD markers like GSK3β and TAU. Vitamin E, in combination with vitamin D, showed a notable increase in GLUT4, p-Akt, and reduced ROS and AD markers. ^51^

**PD**

PD is a progressive neurodegenerative disease, whose pathological conditions can be attributed to the loss of dopaminergic neurons in the midbrain and also to the neuroinflammation caused by neuroglial activation, with clinical abnormalities including motor and non-motor symptoms. A recent study found that both α-T3 and γ-T3 can alleviate neuronal damage caused by 6-OHDA and provide neuroprotection, but it was α-T3 that had a better potential to reduce neuroinflammation. ^16^ The studies conducted by Nakaso *et al.* ^52^ found that γ-T3 and δ-T3 are involved in the activation of PI3K/Akt signaling by binding to Erβ (estrogen receptors) leading to caveola formation when the SH-SY5Y neural cells were subjected to MPP ^+^ toxicity. They have also stated that the antioxidant property of T3 is not responsible for this effect. Later Nakaso *et al*. ^53^ reported that δ-T3 increased motor activities and prevented the loss of neurons in the SN when conducted experiments in MPTP (1-methyl-4-phenyl-1,2,3,6-tetrahydropyridine)-induced C57BL/6 mice. Currently there is an ongoing randomized clinical trial in phase II (NCT04491383), to explore the potential of T3 to slow down the progression of motor and non-motor dysfunctions caused by PD, which was based on previously proven results from experimentation on transgenic animals. This study is being conducted in 100 PD patients who are orally administered with T3 (Tocovid Suprabio (HOV-12020)) or placebo for 104 weeks. ^54^

**Stroke**

An *in vitro* study using cultured HT _4_ hippocampal nerve cells found that, unlike α-Tph, even the nanomolar concentration of α-T3 could suppress the early activation of glutamate-induced c-Src (cellular Src) kinase, by especially blocking glutamate-induced death and thereby saving the neurons. ^55^ Complementing this discovery, another research proved that T3 acts as an important checkpoint to protect against neurodegeneration caused by glutamate-induced cell death and stroke. ^56^

**FRDA**

Another neurodegenerative disease Friedreich’s ataxia (FRDA) is caused due to the downregulation of frataxin, which is a mitochondrial protein. Studies claim that FRDA patients can encounter ferroptosis (caused by impaired mitochondrial failure, lipid peroxidation and glutathione metabolism ultimately leading to iron-dependent cell death). LOX inhibitors such as Tph or T3 [EPI-743 (α-tocotrienol quinone which is now known as vatiquinone or PTC-743) and SFN (sulforaphane) are also Nrf2 inducers], could prevent ferroptosis. Upon study conducted on mouse myoblast with frataxin-gene silenced, cardiac cells of frataxin-KIKO (knockin/knockout) mouse model, fibroblasts of skin and blood of human patients, it was concluded that Nrf2 inducers could neutralize the ferroptosis and that ferroptosis has close correlation with Nrf2 activation. ^57^ EPI-743 is a therapeutic that targets oxidoreductases involved in redox mechanisms. In a randomized placebo-controlled clinical trial (NCT01728064) to determine the clinical effects and safety of EPI-743 in FA patients, it was found that EPI-743 was well tolerated and safe for administration and also that after 24 months of treatment with this drug, a prominent long-term improvement was observed in the neurological function and disease progression. ^58^ Another study performed with five patients who were supplemented a T3 mixture and idebenone found that these patients showed normal results for oxidative stress parameters and other inflammation indices. In addition, they also exhibited an improvement in CMR (cardiac magnetic resonance) readings suggesting a reduction in myocardial damage. ^59^

**VaD**

VaD is the second most common form of dementia, which can be characterized as a gradual drop of cognition. Type 2 diabetes (T2D) patients are at high risk of getting this disease. In the study conducted in T2D rat models, it was concluded that treatment using TRF prevented memory loss and attenuated various biochemical parameters like plasma homocysteine (HCY), blood glucose, SOD levels, acetylcholinesterase (AChE), glutathione (GSH), immunohistochemistry for platelet-derived growth factor-C (PDGF-C). ^60^

### Drug delivery systems

**Bioavailability**

α-TTP (α-tocopherol transfer protein), found in the hepatocytic cytosol, binds the vitamin E administered orally and emphasizes its effective transport between the membranes, identified in rats ^61^ and humans. ^62^ RRR-α-tocopherol is the most biologically active and naturally occurring stereoisomer of α-tocopherol. ^63^ The α-TTP has a higher affinity to this isomer due to the affinity of α-TTP towards the forms of R, ^64^ thus resulting in discrimination. Studies confirmed the existence of bio-discrimination against T3s due to this affinity. ^65^ The absorption of other vitamin E isoforms like γ-T3, if supplemented orally along with α-Tph, will face hindrance because α-TTP binds the α-Tph selectively. ^66^

Studies have shown that T3 has excellent antioxidant activity in *in vivo* systems, but its oral bioavailability is low to rare since they are not identified by the α-TTP. ^67^ According to *in vivo* studies, the plasma concentration of T3 is much lower in the presence of α-Tph. The short t _1/2_ of T3 adds up to its poor bioavailability. ^68^

The highly unsaturated isoprenoid tail contributes to the ability of T3 isomers to cross lipid layers of the brain, liver, and adipose tissue efficiently. γ-T3 in GDT (gamma delta tocotrienol) has been found to have a better bioavailability than TRF, while δ-T3 had a lower bioavailability. A significantly high γ-T3 concentration was found localized in the adipose tissue. ^69^

Vitamin E is absorbed *via* two mechanisms, primarily in the distal portions of the small intestine across the apical enterocyte membrane by passive transport, also assisted by SR-B1, CD36, and NPC1L1 (Niemann-Pick C1-like 1), which are vitamin D transporters. Secondly, through the lymphatic system, as a lipoprotein complex that is composed of bile salt micelles, vitamin E, and chylomicron. Due to its huge size, lipoprotein cannot be transported through the blood capillaries. ^70^

A comparative study of bioavailability and intestinal absorption kinetics, of γ-T3 and α-Tph, both *in vitro* interaction kinetics (in aqueous media) and *in vivo* (in oil media) in rats demonstrated that α-Tph was found to have a significant oral bioavailability (36%) than γ-T3 (9%), which in turn can be concluded based on *in situ* studies, that the α-Tph has a strong intestinal permeability compared to γ-T3. The findings of this study suggest that γ-T3 uptake and bioavailability can be improved by enhancing the solubility and dissolution of γ-T3, leading to its increased permeability. This can be attained either by structural modifications or by exploring new formulation approaches. ^71^

A study conducted in α-TTP-deficient mice proved that α-T3 supplemented orally could restore fertility, and a greater amount of this vitamin was transferred to vital organs. ^55^ Another study demonstrates that α-TTP gene expression in the cultured hepatocytic cells was not substantially necessary for the intracellular localization of α-Tph, γ-Tph, α-T3, and γ-T3. It was also found that neither the methylation of the chromanol ring nor the side chain saturation had any significant role in the distribution of these vitamin E isomers within the cells. These results further indicate the possibility of passive transport, like diffusion, as the key driving force. ^72^ Hence, there could be other mechanisms apart from α-TTP governing the uptake of T3 into the cells.

Other parenteral vitamin E administration modes include intraperitoneal, intravenous, intramuscular, and subcutaneous injections. ^3^

### Nanotechnology-based approaches for enhanced bioavailability

An important strategy to overcome the lower bioavailability of compounds is to opt for nanomedicine, wherein we can take advantage of the small size and greater surface area for effective drug delivery. The preclinical development of T3 nanocarriers comprises niosomes, polymeric NPs (nanoparticles), NEs (nanoemulsions), and NLCs (nanostructured lipid carriers). ^73^ The nanoformulations of hydrophobic/lipophilic compounds or drugs ensure several applications like protection from degradation and enhanced absorption in the GIT (gastrointestinal tract), extended systemic circulation, and regulated drug release. ^3^ Moreover, this technique overcomes the side effects of chemotherapeutic drugs, such as systemic toxicity, and favors accumulation in the specific tumor-infected tissue via target-specific markers to narrow down the possibilities of off-target dispersal. ^74^ It can also improve the half-life, chemical integrity, solubility, and membrane permeability. The interaction of vitamin E with other lipophilic vitamins often leads to its hindered uptake, whereas nanoparticles encapsulating vitamin E can overcome this hurdle. ^70^

NPs usually range in size from 1–100 nm, wherein they either contain drugs distributed evenly within the nanospheres matrix, or a polymer membrane entraps the drug in its cavity (nanocapsules). Novel drug delivery systems are being explored to improvise the pharmacokinetics, bioavailability, stability, and target-specificity of vitamin E, simultaneously by minimizing side effects. ^6^

The encapsulation efficiency of various nanoparticulate systems depends on factors such as the structure of the lipid matrix with respect to crystallinity and the nature of the compound (hydrophilic or lipophilic) to decide lipid compatibility. Since vitamin E is lipophilic, it is compatible with the lipid matrix, assuring higher encapsulation efficiency (EE) and drug loading. These characteristics are quantified to determine the amount of NPs based on the purpose, target, and drug release. Due to its lipophilic nature, vitamin E can be retained in the lipid phase during the NP formation and the majority of the studies claims that it had enhanced EE% (>70%), and even few among them reported values nearly equal to 100%. ^75^

PLGA-Chi (poly-lactic-co-glycolic acid - chitosan) NPs (about 3.5 folds) observed a potentially greater cellular uptake compared to PLGA NPs (both NPs encapsulated with α-Tph and TRF). Both these NPs were able to exhibit antioxidant and antiproliferative properties, and this method can assure greater bioavailability and exploit the pharmaceutical potential of hydrophobic compounds like T3 and Tphs, due to their entrapment abilities. ^76^

**Self-emulsifying drug delivery systems (SEDDS)**

A colloidal dispersion of T3 can be enhanced using SEDDS, which consists of oils, surfactants, and co-solvent. Once introduced into the GIT, the T3 SEDDS becomes a nanoemulsion (100– 300 nm in size) in the presence of gastrointestinal fluids, facilitating *in situ* solubilization of T3. This solubilization aids in the greater surface area for drug release and improves drug partitioning across the enterocytic membrane, thus surpassing the first-pass metabolism via entering the lymphatic system. ^77^

When administered orally, the uptake of T3 becomes limited due to low bioavailability, with <30% of α-T3 and <10% of γ-T3 and δ-T3. ^78^ Due to its lipophilic nature, T3 has poor solubility in the aqueous medium and is a perfect candidate for s-SEDDS to enhance oral bioavailability. These formulations include oils and water-insoluble surfactants, which form fine emulsions or self-emulsions upon exposure to aqueous media and distribute readily in the gastrointestinal tract until it's absorbed. ^79^

*In vivo* study performed in SD rats using s-SEDDS (solid self-emulsifying drug delivery systems) containing 70%, TRF has been shown to effectively ensure better oral bioavailability of α, γ, and δ-T3, with almost similar bioavailability. When the s-SEDDS T3 powder was administered in combination with two different surfactants, namely poloxamer and Labrasol ^®^, this formulation exhibited a faster absorption rate than that of formulations with only one of these surfactants and also non-self-emulsifying liquid TRF. ^80^

Studies have shown that by incorporating γ-T3 in the formulation of a self-emulsifying drug delivery system (SEDDS), its solubilization and passive permeability were significantly enhanced, therefore, improving its oral bioavailability and intracellular absorption compared to the Tocovid (a commercially available form of T3). ^81^

γ-T3, with its unsaturated phytyl chain, can easily enter tissues, ultimately resulting in an even distribution in the lipid bilayer of the plasma membrane. Its tissue uptake is assumed to be facilitated by receptor-mediated lipoprotein endocytosis and aided by lipoprotein lipases. The γ-T3 evenly distributed in the membrane interacts with lipid radicals using its chromanol head, readily eliminating peroxyl radicals. ^82^

**Lipid-based nanoencapsulation methods**

Nanoencapsulation is a method to improve bioavailability where a matrix (secondary or shell material) entraps or encapsulates bioactive core materials, forming nanocapsules together. This drug delivery technique can be implemented to deliver oil-phase or water-phase successfully, prepared using emulsifiers, co-solvents, and carrier substances. The core material includes vitamins, minerals, lipids, enzymes, antioxidants ( *e.g.* terpene, black pepper, and limonene), probiotics (such as *Lactobacillus*, and *Bifidobacteria*), *etc.* The matrix is usually constituted by lipids, cellulose, gum (gum acacia, gum arabic), *etc.* Modified polysaccharides are being used in the nanoencapsulation process of target molecules, assisted by various fabrication techniques like supercritical fluid, electrospray, spray drying, reverse micelle, electrospinning, coacervation, *etc.* ^83^

Lipid-based nanoencapsulation has low toxicity, greater encapsulation efficiency (EE%), and can be produced at an industrial scale. Owing to the amphiphilic nature of these NPs, this technique also serves the advantage of encapsulating hydrophilic and lipophilic compounds with different polarities simultaneously, such as polyphenols, flavonoids, fatty acids, and carotenoids. ^84^

**Liposomes and nanoliposomes**

The sub-micron bilayer lipid vesicles or nanoliposomes are colloidal vesicles that entrap bioactive compounds and deliver them to the target site, enhancing bioavailability, reducing toxicity, and side effects, controlling the release of the drug, extending shelf life, *etc.* ^83^

Curcumin extracted from the plant *Curcuma longa* is a lipophilic phytochemical with numerous bioactive properties such as antioxidant, antimicrobial, anti-inflammatory, and anti-tumorigenic. Even though curcumin has the least oral toxicity and highest bioactivities compared to its curcuminoid counterparts, its hydrophobic nature, and poor water solubility results in reduced oral bioavailability. ^85^ The curcumin-loaded nanoliposomes were found to have improved stability, physicochemical properties, controlled drug release properties, and equivalent cellular antioxidant activity (CAA). But the curcumin entrapped in nanoliposomes had lower cellular uptake than the free curcumin. ^86^ It was found that with surface modifications of liposomes, they can be directed to treat neuro-diseases as possible phytochemical carriers. The curcumin-loaded liposome conjugated with sialic acid and wheat germ agglutinin exhibited an enhanced encapsulation efficiency and permeability through the monolayered endothelial cells in the BBB. ^87^

**Nanoemulsion**

Nanoemulsions (NE) are formed by two immiscible phases (oil and water, either o/w or w/o) in the presence of one or more surfactants. These kinetically stable dispersions can be prepared through various techniques like microfluidization, high-pressure homogenization, and phase inversion temperature emulsification (PIT). These recent methods have overcome the drawbacks posed by conventional modes, such as sedimentation and creaming, producing NE with prolonged colloidal stability and low viscosity. High-pressure homogenization aided in the characterization of vitamin E nanoemulsions, wherein the lipids were digested rapidly, ensuring improved bioaccessibility, due to their smaller droplet size (80–300 nm). ^3^ The characteristics that influence the performance of NE are zeta potential, droplet size, and drug content. ^88^ NE approach has been used recently for controlled drug release of biologically active compounds using oil, water, and surfactants to give a colloidal nanosized dispersion. This NE system has many advantages, including increased emulsion stability, bioavailability, antioxidant effects, and altered texture. There are several NEs, namely, pickering NE, single NE, double NE, and structural NE (with the interfacial layer being single or double). ^83^

Encapsulation within a carrier increases the brain’s uptake of curcumin. For example, curcumin NE ^89^ and curcumin-docosahexaenoic acid microemulsion (ME) enhanced the impact of the drug on malignant glioblastoma neural cells U-87. ^74^ This method can ensure the uptake of drugs *via* lymphatic transportation, thus increasing the bioavailability of hydrophobic drugs.

**Solid lipid nanoparticles (SLN)**

SLNs are nanocolloidal drug delivery systems (50–1000 nm) composed of lipids, drugs, and surfactants in specific proportions. These are spherical solid structures that remain intact at room and body temperatures. It involves surfactants to stabilize the solid lipid cores. The core materials include fatty acids, acylglycerol, waxes, *etc.* while stabilizers involve cholesterols, phospholipids, and sphingomyelins. SLNs are used as carriers for drug encapsulation, to successfully deliver antioxidants like quercetin, curcumin, bixin, puerarin, *etc.* for therapeutic purposes. ^83^

These nanostructures have several benefits, including prolonged stability, site-specific targeting, shielding labile drugs, controlled drug release, transporting both hydrophilic and lipophilic compounds, non-toxicity, ease of preparation, and cost-effectiveness. Contrary to this, SLNs pose a few drawbacks, such as a moderate drug expulsion rate due to their crystallization (under storage conditions) and limited drug loading ability. ^74^ A hydrophobic drug, indirubin, used in ancient Chinese medicine, was loaded in SLNs to target U87MG human glioblastoma cells and was found to have enhanced the anticancer activity of this lipophilic compound. ^90^

**Nanostructured lipid carriers (NLCs)**

NLCs are second-generation lipid-based nanocarriers designed to overcome the limitations of SLNs. The NLCs can carry lipophilic and hydrophilic compounds, be oriented to site-specific targets, surface modified, controlled drug release, and have poor *in vivo* toxicity. Since liquid lipids are present in the nanoformulation of the NLCs, it prevented lipid crystallization that greatly decreased drug expulsion, which earlier challenged their storage. ^74^ Additionally, it has improved drug loading capacity ^74^ and shields the bioactive compounds from enzymatic and biological degradation. Lipid-based nanocarriers involving solid lipids mixed with liquid lipids are usually prepared by mixing both solid and liquid lipids in a ratio of 70:30. ^83^

NLCs can improve absorption through the GIT, surpassing hepatic first-pass metabolism, by entering into the mesenteric lymphatic system via M cells. P-gp (P-glycoprotein) efflux inhibition by the surfactants present on the shell of NLCs facilitates their uptake. Some lipophilic bioactive can be added to enhance GI transport. The bioavailability of hydrophobic molecule coenzyme Q10 was improved using NLC as the carrier. ^70^ Baicalein-loaded tocol NLCs (tocol NLCs are composed of vitamin E, phospholipids, tripalmitin, gelucires, and poloxamer 188) was administered intravenously, and the vitamin E in the NLC contributed to its *in vivo* stability with prolonged half-life and enhanced the brain penetrating efficiency of baicalein. Profound amounts of baicalein were found in various parts of the brain, especially the cortex, brain stem, hippocampus, thalamus, striatum, and olfactory tract, when NLC was used as a carrier. ^91^

**Quantum dots**

Nanotheranostics, a technique used for therapeutic and diagnostic purposes, might overcome the challenges BBB poses. This method focuses on drug delivery systems based on quantum dots (QDs), which are zero-dimensional semiconductor nanocrystals (2–10 nm). QDs serve the advantage of having the ability to cross BBB and, due to their biocompatibility towards neurons, have low toxicity and can be oriented to target neurodegenerative diseases. Its intermediary size between discrete molecules and bulk semiconductors increases its efficiency in diagnosing and treating, mainly owing to its remarkable electrochemical and optical properties. These nanostructures possess certain distinguishing characteristics such as electronic properties, photostability, luminescence, size-tunable emission, high excitation capacity, and others, which conserve their capabilities for diagnosis, therapeutics, biosensing, and bioimaging. ^92^ Curcumin QDs exhibited a greater potential in degrading bacteria biofilms than curcumin alone. Few QDs, like chlorophyllin and folic acid, are found to be potential candidates for imaging diagnosis. ^93^ This opens possibilities for targeting T3 as QD to treat neurodegenerative diseases.

**Challenges while targeting the brain**

The oral administration usually requires a heavy dosage due to systemic circulation and hepatic first-pass metabolism. Recent studies have shown that the nasal-brain route can be focused to deliver nanocarriers for drug administration as it can bypass the BBB and this may be a potential route if some of the drawbacks, such as rapid mucociliary clearance, enzymatic degradation, and low permeation rate of drug through the nasal epithelium are addressed. The CNS has barriers like BBB and BCSFB (Blood cerebrospinal fluid barrier) to shield it from the entry of neurotoxic xenobiotics and peripheral blood circulation. The BBB includes tight junctions, multidrug efflux pumps, protein transporters, enzymatic barriers, and others, while BCSFB includes tight junctions joining the choroid plexus epithelium. The physicochemical nature of the compound, such as lipophilicity and molecular weight, are the key factors influencing the transport *via* BBB. ^94^

### Why target the nose–brain route for neurodegenerative diseases?

The intranasal route is non-invasive, has quick therapeutic delivery, and surpasses the first-pass metabolism and the BBB. ^94^ Several *in vivo* studies prove that drug administration through the nasal epithelium can induce a better impact than any parenteral delivery method, including intravenous. Due to its mucoadhesive and penetration-enhancing ability, chitosan when incorporated into NEs, has exhibited high permeation and flux via the nasal membrane, compared to the NEs devoid of chitosan. The NEs gained an inherent mucoadhesive ability when formulated with chitosan. ^88^

On intranasal (IN) administration, the drug’s nanodroplets are transported through the endothelium in the brain via endocytosis or transcytosis mechanisms. The drug penetrating ability is amplified by the surfactants contained in the NE, which has a fluidizing impact on the endothelium. ^88^ The contact time in the nasal epithelium can be prolonged by inhibiting the P-gp efflux pump, which ultimately increases the intranasal administration to the brain. ^95^

Intranasal supplementation of selegiline (lipophilic) NE enhances its bioavailability to the brain due to safe-guarding from degrading enzymes in the nasal epithelium, bypassing the first-pass metabolism and systemic circulation. After this intranasal treatment in rats, it almost normalized dopamine levels along with restoration of non-enzymatic and enzymatic markers of lipid peroxidation and reverted the haloperidol effect leading to regaining the cellular integrity. Thus, intranasal delivery of selegiline was more promising since it can mend dopamine levels and antioxidant scarcity and target the CNS without crossing the BBB. ^96^

A curcumin-loaded NLC prepared using the hot high-pressure homogenization method was found to have anticancer effects and enhanced drug entrapment and release properties. X-ray diffraction (XRD) and Differential scanning calorimetry (DSC) analysis revealed that the NLCs contained the drug in an amorphous form. Following this drug administration, there was a noticeable increase of curcumin in the CNS, and also the NLC exhibited very low toxicity towards astrocytoma-glioblastoma cells and is biocompatible and biodegradable. Thus, this study concludes that IN mode of drug delivery can be an alternative route for therapeutic applications to treat neuro-diseases, including glioblastoma. ^97^

## Future prospects

Recently, intranasal (IN) drug administration has been of particular interest to researchers since it is non-invasive, ensures greater bioavailability and offers options for a low dosage of drugs. Even though this route has many advantages, its challenges are quite difficult to overcome. More studies are to be conducted to understand the mechanism, bioavailability, uptake, and therapeutic action of T3 when targeting the brain via this route. Since nanomedicine can enhance the bioavailability of the drug, there is a wider possibility to explore the various nanocarrier methods for effectively transporting T3 to treat neurodegenerative diseases. Different combinations of lipid carriers, surfactants, and mucoadhesive polymers can be chosen based on the targeted disease condition. Since few phytochemicals when used as quantum dot candidates exhibited amplified bioactive properties, this opens possibilities to explore similar strategies for tocotrienol as a worthy candidate. If tocotrienol QD is quantified and characterized, then it can be directed toward targeting neurodegenerative diseases, which could aid in early diagnosis and treatment.

## Conclusions

Despite being a promising candidate with numerous therapeutic applications, T3 could not be exploited to its full potential owing to its poor oral bioavailability. Its anti-inflammatory property is of particular interest since it can be used to target progressive neurodegenerative diseases, which are usually fatal. New possible drug delivery techniques based on nanotechnology offer options to target T3 towards the brain to attenuate neurodegenerative diseases. But the characterization and quantification of T3 nanocarriers targeting the brain are yet to be proven. Successful nano-drug delivery methods of other lipophilic bioactive compounds have been discussed in this review to highlight the possibility of using similar formulations for T3. The hepatic first-pass metabolism and BBB pose serious threats to focusing the CNS (central nervous system) to treat neuro-diseases. The nose-brain route rules out this troubleshooting and allows for low dosage and lesser side effects. Intranasal administration is a promising method for T3 due to its lipophilicity and low molecular weight, more research can be focused in this arena.

## Data Availability

No data are associated with this article.
