# Supplementary material for: Exploring the anti-inflammatory activities, mechanism of action and prospective drug delivery systems of tocotrienol to target neurodegenerative diseases
Source: F1000Res. 2023 Mar 27;12:338. [Version 1] doi: 10.12688/f1000research.131863.1 (PMC11406131; doi:10.12688/f1000research.131863.1)
Supplement: Peer review history

# Reviewer report: Reviewer response for version 1

**DOI:** 10.5256/f1000research.144748.r204573

**Published:** 2024-09-16

**Authors:** Rahul Shukla

**Affiliations:** 1 National Institute of Pharmaceutical Education and Research-Raebareli,, Lucknow, India

The article, " *Exploring the anti-inflammatory activities, mechanism of action and prospective drug delivery systems of tocotrienol to target neurodegenerative diseases*" is well-written. The paper is an important addition to the literature.

 They can further read various articles on neurodegenerative disorders in recent years relevant to drug delivery.

 There are a few grammatical mistakes in the manuscript, could you correct these errors for clarity of content?

 More examples need to be incorporated in the manuscript in various delivery systems.

 It can be indexed after minor revision.

Is the review written in accessible language?

Yes

Are all factual statements correct and adequately supported by citations?

Partly

Are the conclusions drawn appropriate in the context of the current research literature?

Yes

Is the topic of the review discussed comprehensively in the context of the current literature?

Partly

Reviewer Expertise:

Drug Delivery Systems for Neurodegenerative disorders and cancer

I confirm that I have read this submission and believe that I have an appropriate level of expertise to confirm that it is of an acceptable scientific standard.

# Reviewer report: Reviewer response for version 1

**DOI:** 10.5256/f1000research.144748.r204574

**Published:** 2023-09-19

**Authors:** Arunachalam Muthuraman

**Affiliations:** 1 AIMST University, Semeling, Malaysia

**Overall comments:** Mathew *et al. *described in this manuscript: the role of tocotrienol drug delivery pattern for the exploration of anti-inflammatory actions for the management of neurodegenerative diseases. The authors also described the pharmacokinetic profile for the exploration of tocotrienol drug delivery techniques. The major limitation is manuscript has expressed mixed action of T3 on neurodegenerative and non-neurodegenerative disorders. Multiple statements are vague. The overall manuscript needs to improve and it may help in a better understanding, of those working in this field of research.

**Specific comments:**

1. Multiple sections like Introduction, anti-inflammatory properties, etc. have too many small paragraphs. Need to be concise with limited paragraphs with a logical sequence of the statements.
2. Figure 2. The target must be cited as evidence of T3 actions. Need to highlight based on actions on neurodegenerative disease.
3. Figure 3. The tag created in Biorender.com can be removed; can be mentioned in figure legends.
4. Each neurodegenerative section end needs to conclude the T3 action on respective neurodegenerative disorders.
5. Table 2 references can be placed as the last column.
6. T3 action on VaD is covered limited; needs to expand similarly to other disorders.
7. The consequences and challenges of T3 action for neurodegenerative disorders and the importance of Drug delivery systems application for T3 therapy need to be explained before the bioavailability section.
8. The statement ‘Other parenteral vitamin E administration modes include intraperitoneal, intravenous, intramuscular, and subcutaneous injections.3’ is incomplete hanging. The various route-based PK profiles of T3 must be discussed.
9. What is the need for Curcumin discussion in the Liposomes and nanoliposomes formulations?
10. Future directions and conclusion author emphasized the nano-delivery of intranasal administration T3. These sections must cover all other nano-delivery concepts or the title must be changed based on the intranasal nano-delivery systems.

**Minor comments**

1. Should be reducing the appearance of too many small paragraphs.
2. Text alignment and typo errors need to be rectified.

Is the review written in accessible language?

Yes

Are all factual statements correct and adequately supported by citations?

Partly

Are the conclusions drawn appropriate in the context of the current research literature?

Partly

Is the topic of the review discussed comprehensively in the context of the current literature?

Yes

Reviewer Expertise:

Neuropharmacology.

I confirm that I have read this submission and believe that I have an appropriate level of expertise to confirm that it is of an acceptable scientific standard, however I have significant reservations, as outlined above.

# Reviewer report: Reviewer response for version 1

**DOI:** 10.5256/f1000research.144748.r188254

**Published:** 2023-08-03

**Authors:** Abhijnya Kanugovi

**Affiliations:** 1 Stanford University, Stanford, California, USA

The article clearly compiles all the relevant literature for using tocotrienol as a drug to target neurodegenerative diseases. The article clearly describes the medicinal values of Tocotrienol, and the problems encountered in drug delivery. While addressing the problem of Tocotrienol delivery, the authors clearly describe the existing methods and their shortcomings and suggest strategies to solve the issue. In summary, the review article is very comprehensive for researchers studying in this field.

Is the review written in accessible language?

Yes

Are all factual statements correct and adequately supported by citations?

Yes

Are the conclusions drawn appropriate in the context of the current research literature?

Yes

Is the topic of the review discussed comprehensively in the context of the current literature?

Yes

Reviewer Expertise:

Cell biology, Therapeutics, Stem cells, Neurology, Cancer.

I confirm that I have read this submission and believe that I have an appropriate level of expertise to confirm that it is of an acceptable scientific standard.
